# Unstable Delamination Growth in Stiffened Composite Panels Under Cyclic Loading Conditions

**DOI:** 10.3390/polym16223118

**Published:** 2024-11-07

**Authors:** Rossana Castaldo, Angela Russo, Mauro Zarrelli, Cinzia Toscano, Aniello Riccio

**Affiliations:** 1 Department of Engineering, University of Campania “Luigi Vanvitelli”, Via Roma 29, 81031 Aversa, Italy; angela.russo@unicampania.it (A.R.); aniello.riccio@unicampania.it (A.R.); 2 IPCB Institute of Polymers, Composites and Biomaterials, CNR National Research Council, Piazza Granatello, 80055 Portici, Italy; mauro.zarrelli@cnr.it; 3 CIRA, Centro Italiano Ricerche Aerospaziali, 81043 Capua, Italy; c.toscano@cira.it

**Keywords:** delamination, Paris law, stiffened composite panel, unstable growth

## Abstract

Aeronautical structures can be damaged by objects during operation and maintenance. Indeed, foreign object impacts (FOIs) affect the overall performance of composite structural components. Delamination is the most critical damage mechanism as it is undetectable and develops silently. This phenomenon can be worsened by cyclic loading, as residual strength and stiffness can decrease rapidly, potentially leading to collapse. Unstable delamination growth is critical because it can occur without an increase in the applied load, threatening the integrity of the structure. Predicting this behaviour under fatigue loading is challenging for standard non-linear finite element methods (FEMs), which often face convergence problems when simulating the dynamic nature of delamination growth. This paper presents an efficient alternative methodology for analysing the propagation of delamination under cyclic loading in composite structures, with non-linear static analyses. This new methodology has been shown to be able to correctly account for the decrease in load carrying capacity during growth by performing ad hoc iterations with alternating force and displacement-controlled FEM simulations. To achieve this objective, the Paris law approach has been implemented in the ANSYS FEM code together with an enhanced virtual crack closure technique (VCCT)-based method. The model correctly predicted delamination growth in stiffened aeronautical panels with artificial delaminations subjected to cyclic compression loading.

## 1. Introduction

In recent decades, studies of the properties of composite materials have revealed their excellent mechanical characteristics, including high specific stiffness, specific strength, and reliability under fatigue loading conditions. These advantages have led to a significant increase in their use in the aeronautics industry for thin-walled composite structures, such as wings, tail planes, and fuselage, where the stringer is bonded or co-cured to the skin. Despite their many advantages, composite laminates are susceptible to damage caused by impact during maintenance or operation, improper curing of composite materials, inadequate adhesion of composite layers, or material exposure to high temperatures. The main damage that occurs in these structures is delamination, which is the failure of the interface between two layers, mainly due to the lack of reinforcement in the thickness direction. This damage is often undetectable by visual inspections and can develop unstably and silently within the component [1,2,3,4]. 

Delamination can change the strength and stiffness of the structure by altering its load carrying capacity, particularly when subjected to static and fatigue compressive loading conditions. The loading/unloading process, during fatigue cycles, can result in an increase in delamination and decrease in the residual strength and stiffness, which can compromise the load-carrying capability and lead to structural collapse. In some cases, depending on the geometry and material characteristics, the propagation of the delamination may become unstable, leading to a significant growth without the need to increase the external load. It has been established by researchers that delamination under fatigue loading is frequently initiated by microscopic defects or stress concentrations, such as those situated in the vicinity of the edges of composite laminates or around fasteners. It has been demonstrated in studies that factors such as the orientation of fibres, the properties of the matrix, and the external environment (e.g., temperature and humidity) can have a significant influence on the growth of delamination during fatigue [5,6,7,8,9,10,11,12]. The numerical simulation of delamination growth is based on the principles of fracture mechanics, especially the energy release rate (G), which represents the rate at which energy is released during delamination growth. Typically, the VCCT (virtual crack closure technique) and the linear law criterion are employed to simulate the delamination growth phenomenon by comparing the energy release rates with their critical values [13,14]. In simulations of fatigue loading conditions, a sequence of static analyses with force control, together with a Paris law approach, is employed to consider the propagation of delaminations. Nevertheless, this approach may fail for unstable delamination propagation in structures exhibiting both local (delamination) and global elastic instabilities. This is due to the convergence issues associated with the highly dynamic behaviour of unstably growing delaminations and to the local and global buckling phenomena. The objective of this research is to develop an efficient numerical methodology that can effectively simulate the unstable propagation of delamination under cyclic loading conditions in composite material structures. The real added value of this study is the use of the FT-SMXB numerical tool, which is able to overcome the limits in terms of mesh and load step dependency of the standard VCCT implemented in the commercial FEM software Ansys 18.0. Such a numerical procedure has been validated for both static and fatigue analyses on both simple and complex structures, as evidenced in references [15,16,17,18]. This methodology allows for the examination of rapid fluctuations in delamination size, as well as the reduction in the structure’s bearing capacity that occurs during the unstable growth phenomenon. The new methodology employs a sequence of non-linear static analyses with applied displacements and reaction force control, with the objective of aligning the predicted level of the load with that indicated by the fatigue module. The number of cycles where delamination growth is expected to occur is evaluated using Paris’ law [19,20]. This methodology has been implemented in the ANSYS FEM code by using the fully parametric APDL language.

The developed numerical model has been demonstrated to accurately predict the unstable delamination growth in aeronautical stiffened panels with artificial circular delaminations of varying depths and sizes, subjected to cyclic compression loading. The proposed novel approach is fully described in Section 2, while the test case description of the stiffened composite panels is detailed in Section 3. The results and conclusions are reported in Section 4 and Section 5, respectively.

## 2. Novel Fatigue Numerical Approach

The novel fatigue numerical approach uses a sequence of non-linear static analyses performed according to the VCCT (virtual crack closure technique), which allows for the evaluation of the energy release rate (1):

(1) $$G_{j}=\frac{F_{j}∆u_{j}}{2∆A}\;\;\;\;\;with\;j=I,II,III$$

where $F_{j}$ is the load on the surface of the crack length, $∆u_{j}$ is the crack opening displacement, ∆A is the variation crack length, with force and displacement associated to the crack mode j (mode I is referred as the interlaminar tension mode, mode II is referred as the interlaminar sliding shear mode and mode III is referred as the interlaminar scissoring shear mode). The fatigue evolution crack is predicted by utilizing the VCCT method together with the Paris law, which relates the growth of delamination (a) for each single cycle (N) to a function of the energy release rate (G), as shown in Equation (2):

(2) $$\frac{da}{dN}=f(G)$$

In Equations (3)–(6), different crack growth rate equations are introduced. These can be selected by the user based on their needs and specific applications.

(3) $$\frac{da}{dN}=c_{1}{\left(G_{max}\right)}^{c_{2}}$$

(4) $$\frac{da}{dN}=c_{1}{\left(G_{max}-G_{min}\right)}^{c_{2}}$$

(5) $$\frac{da}{dN}=c_{1}{\left[{\left(\sqrt{G_{max}}-\sqrt{G_{min}}\right)}^{2}\right]}^{c_{2}}$$

(6) $$\frac{da}{dN}=c_{1}{\left(\frac{G_{I}}{G_{Ic}}\right)}^{c_{2}}+c_{3}{\left(\frac{G_{II}}{G_{IIc}}\right)}^{c_{4}}$$

According to Equations (3)–(6), $G_{max}$ and $G_{min}$ represent the strain energy release rate (ERR) values associated with the maximum and minimum applied loads, respectively. The parameters *c*_1_, *c*_2_, *c*_3_, and *_C_*_4_ are experimentally determined fitting parameters. $G_{I}$ and $G_{II}$ denote the ERR values corresponding to mode I and mode II delamination, while $G_{Ic}$ and $G_{IIc}$ indicate their critical values.

The fatigue analysis imports the ERR components from the VCCT analysis at the conclusion of each individual non-linear static analysis (where the selected force is reached). Subsequently, the number of cycles are calculated in accordance with the parameters set forth in Equation (7), wherein the increment in the delaminated area at the specified cycle, designated as $∆A_{i}^{k}$, is taken into account.

(7) $$∆N_{i}^{k}=\frac{∆A_{i}^{k}}{f\left(G\right)}$$

It can be seen that the node with the lowest value of $∆N_{i}^{k}\;$(maximum value of f(G)) is selected and denoted as j. Following this, the damage vector is calculated using Equations (8) and (9), with components related to each delamination front node. The t position in which the damage vector is maximum corresponds to the disconnected node, and the node is detached, with $Damage_{t}^{k}$ reset to 0.

(8) $$∆{Damage}_{i}^{k}=\frac{∆N_{j}^{k}}{∆N_{i}^{k}}(1-{Damage}_{k}^{k-1})$$

(9) $$Damage_{i}^{k}=Damage_{i}^{k-1}+∆{Damage}_{i}^{k}$$

Subsequently, the number of fatigue cycles are updated, as described in Equation (10), and the procedure is iterated for the subsequent cycle k + 1.

(10) $$N_{i}^{k}=N_{i}^{k-1}+∆N_{j}^{k}(1-{Damage}_{t}^{k-1})$$

**Figure 1:**
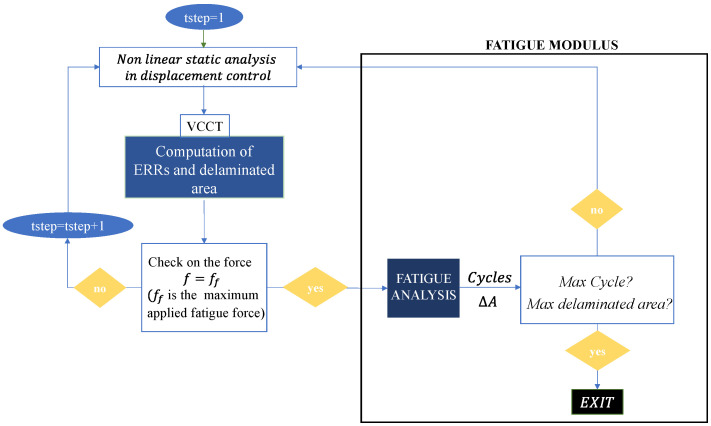
Novel fatigue loop method.

Figure 1 shows a flowchart illustrating the recently implemented methodology. In order to analyse a specific cycle, it is first necessary to subject the structural composite model, which has been previously damaged, to the appropriate boundary conditions and then perform a non-linear static FEM analysis under displacement control. The VCCT method allows for the evaluation of both the increasing delaminated area and the energy release rate. Once the non-linear analysis is complete, a check is made on the reaction force; if the reaction forces are equal to the applied forces during a fatigue cycle, the iterations are terminated. Otherwise, the applied displacements are increased and a new check on the resulting reaction force is performed. Once the reaction forces are equal to the applied forces ($F_{f})$ during the fatigue cycle, the information from the non-linear static analysis, in terms of energy release rates, is transferred to the fatigue modulus. This, in conjunction with the Paris law equations, allows us to evaluate the number of cycles at which the delaminated area increases. A new cycle is initiated from this point, and this process continues until the maximum number of cycles is reached.

## 3. Numerical Application

**Figure 2:**
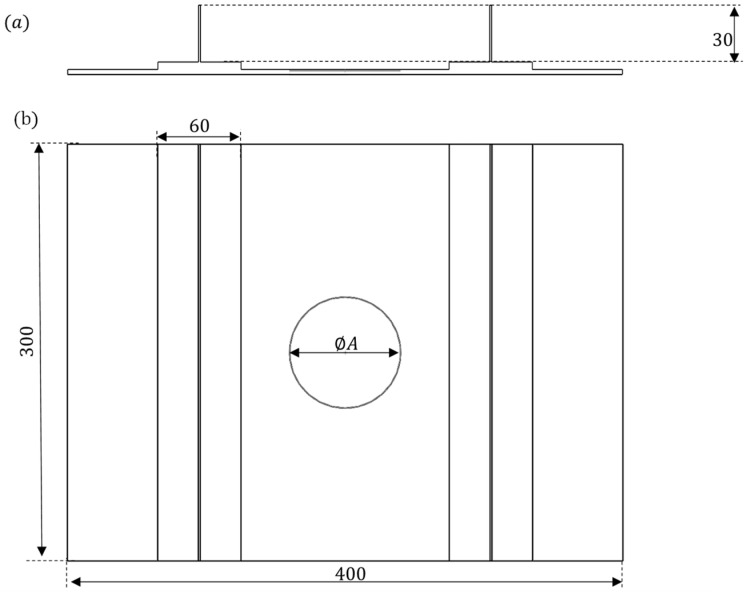
Double-T-stringers composite specimen (all dimensions have millimetres units). (**a**) Cross-section view; (**b**) frontal view.

As previously stated, the novel developed methodology has been implemented in order to assess its efficacy. It has been applied to a stiffened composite panel with delamination under compression–compression fatigue loading conditions. Figure 2 illustrates the graphical representation of the numerical test case used to evaluate the unstable propagation of delamination in composite structures. The test case serves as a benchmark for a typical aeronautical composite-stiffened panel with T-shaped stringers and a pre-existing artificial embedded delamination in the central bay. A variety of configurations have been contemplated, distinguished by different values of the delamination diameter (A) and depth.

In order to investigate the influence of geometrical parameters on the unstable growth of delaminations, different values of the diameter and of the depth of the delamination have been considered. All the other geometric parameters of the panel, as illustrated in Figure 2, have been unchanged (skin: 300 × 400 mm, stringers foot: 60 mm, and stringer web: 30 mm).

**Figure 3:**
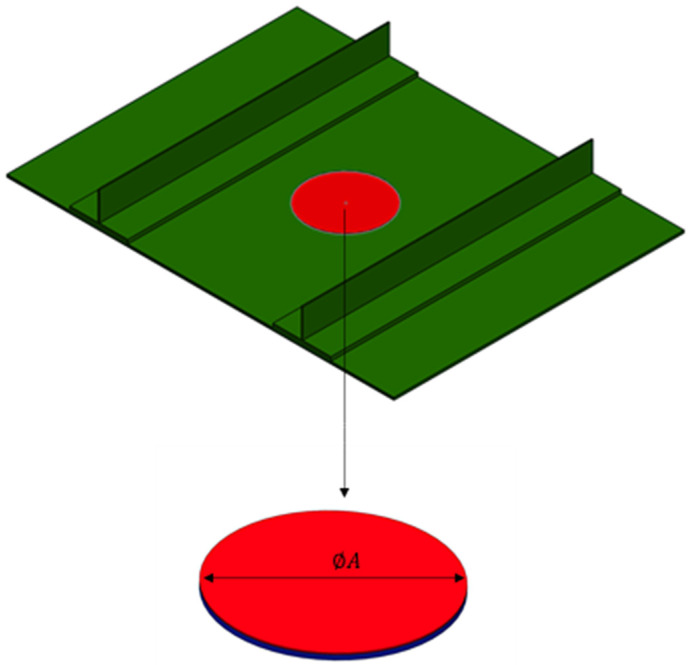
Double-T-stringers composite specimen.

The stacking sequence of the skin is [−45, 90, 0, 45] 2s while the stringer foot and the stringer web have a [0, 90, 90, 0] s layup. The thickness of each layer is 0.165 mm. The geometric dimensions have been chosen according to the stiffened panels investigated in [15]. Figure 3 depicts the solid model of the stiffened composite panel with the artificial circular delamination highlighted in red.

**Figure 4:**
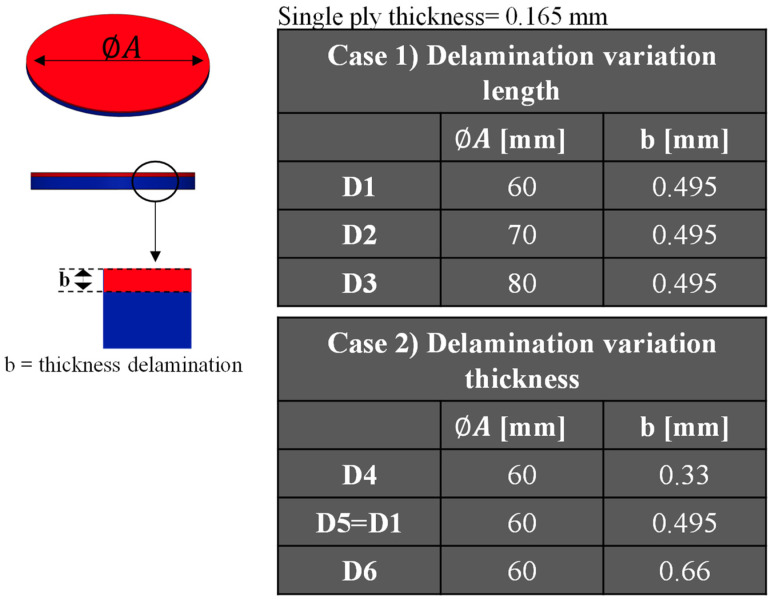
Delamination diameters and thickness. Case (1) delamination variation length. Case (2) delamination variation thickness.

Six configurations have been analysed, as showed in Figure 4, with varying diameters and depths of the delamination. The D1, D2, and D3 configurations are characterised by a uniform delamination depth of 0.495 mm, while exhibiting disparate delamination diameters. Configuration D1 is characterised by a delamination diameter of 60 mm, configuration D2 by a delamination diameter of 70 mm, and configuration D3 by a delamination diameter of 80 mm. The remaining three configurations, indicated as D4, D5, and D6, are characterised by the same delamination diameter of 60 mm and different delamination depths. The delamination depth of configuration D4 is 0.33 mm, while that of configuration D5 is 0.495 mm. Finally, configuration D6 has a delamination depth of 0.66 mm.

The panel was made by using a carbon fibre/epoxy resin material system, and its properties were assessed through an experimental campaign following the established ASTM test standards. The tests were conducted at the National Research Council of Italy, employing an MTS test machine. The mechanical properties derived from these evaluations are detailed in Table 1.

### FEM Model

**Figure 5:**
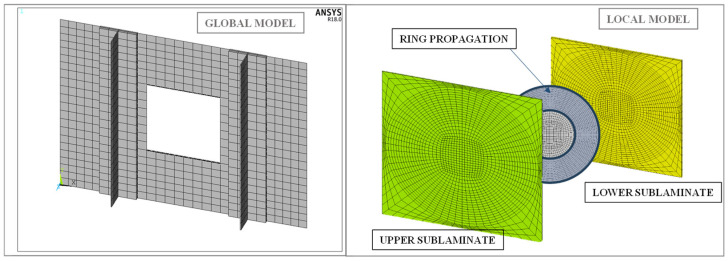
Fem model—global and local fem model.

A parametric approach was employed using Ansys 18.0 software to construct the finite element models of the composite panels with two T-Shaped stringers and delaminations. In order to optimise the computational efforts, a global–local approach has been adopted for the delaminated area. In accordance with the global–local approach, a coarse mesh has been employed to represent the overall panel (global model), whereas a detailed mesh has been used for the delaminated region and the anticipated propagation area (local model). The global and local models have been connected through contact elements with multipoint constraints. The region containing the circular delamination has been modelled through two sublaminates: an upper and a lower one, joined by contact elements with life and death capabilities. These elements are strategically positioned on the ring of the delamination, where damage propagation is expected to take place, as illustrated in Figure 5.

The nodes of the local model boundaries have been connected to the nodes of the global model via contact elements with multipoint constraints. The discretization process employed a hybrid combination of solid and shell elements. In particular, SOLID186 elements, characterized by 20 nodes with three degrees of freedom each, have been selected for the stringers, the underlying skin, the delaminated area, and the propagation zone. The layered structural solid option was configured in order to ensure the accurate allocation of composite materials and stacking sequences. In the remaining portion of the panel, layered shell elements have been employed. The model developed is parametric, allowing the discretisation to be adapted to the dimensions of the panel. Specifically, the global model was discretised using an average element size of 13 mm in both length and width. In contrast, the local model has variable element sizes, with a refined mesh concentrated in the area of the initial artificial delamination. In addition, the mesh density of the local model is configured at 7680 elements per 38 cm^3^;, which improves the accuracy of the analysis by ensuring a detailed representation of the delamination effects in critical regions. The behaviour of composite-stiffened panels with two T-stringers and a circular embedded delamination has been investigated under both static and fatigue loading conditions. An initial non-linear static analysis under displacement control was conducted using SMXB [17,18] to determine the stiffness and strength of the structure. Furthermore, the static onset delamination load was identified through this analysis. In light of these preliminary findings, the load to be applied under fatigue has been selected. In both analyses, the appropriate boundary conditions were implemented. On one side of the panels, rotational and translational degrees of freedom were completely suppressed, while on the other side, axial compressive displacement was applied.

## 4. Numerical Results and Discussion

In this section, the results of numerical analyses performed on conducted configurations are introduced. Initially, the behaviour of stiffened composite panels was investigated through non-linear static analyses, which varied the diameter and depth of delamination. The stiffness, static onset delamination loads, and local delamination buckling loads were determined. Subsequently, all configurations were subjected to cyclic loads in order to determine the unstable delamination growth. The diameter of the delamination was a particular focus of attention at various points during this process.

### 4.1. Effects of Delamination Diameter (Non-Linear Static Analyses)

The D1, D2, and D3 configurations have identical geometries with the sole distinction being the initial damage diameter, which is 60 mm, 70 mm, and 80 mm, respectively. Table 2 presents the stiffness values for the three panels, which are found to be equal, thereby confirming that the diameter of the damage does not influence the global stiffness. Subsequently, these configurations were subjected to non-linear static analyses, with out-of-plane displacements calculated at the centre of circular delamination. The instability loads, created by bending, are presented in Table 2. The Euler formula is a mathematical expression that is used to calculate the critical load of the panel under compression. It states that the buckling load is inversely proportional to the square of the free inflection length. Consequently, the critical load of deflection decreases as the free length of deflection increases. The results obtained from the conducted analyses confirm this trend. The D1 configuration, which has a diameter damage of 60 mm, has a buckling damage load of 47,351 N; the D2 configuration has a load of 34,129 N; and the D3 configuration, which has the largest diameter, has a load of 28,000 N.

**Figure 6:**
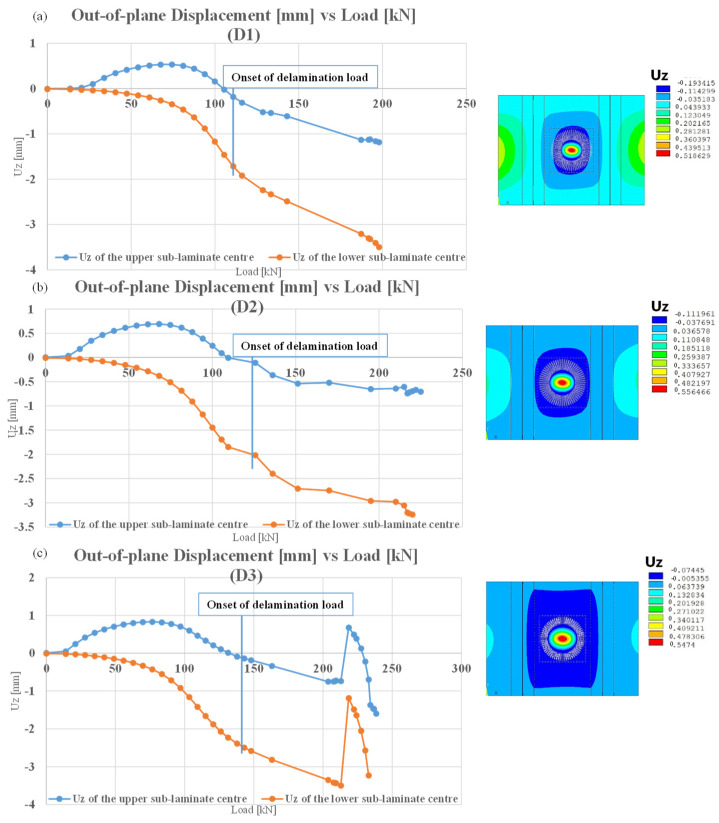
Out of plane displacement versus load for D1, D2, and D3 configurations.

**Figure 7:**
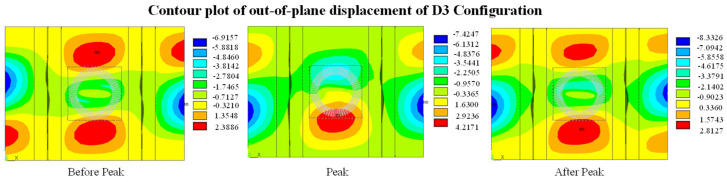
Contour plot of out of plane displacement of D3 configuration.

Figure 6 illustrates the qualitative performance of the out-of-plane displacements in the centre of the circular delamination. The calculations are presented for the centre of the first layer of the upper laminate and on the last layer of the bottom laminate. Figure 6 illustrates that panel D3 exhibits a different buckling behaviour compared to the other two panels. Indeed, after 200 kN, the panel undergoes three changes in buckling modes, which correspond to the three moments: prior to the peak, during the peak, and after the peak of the curve, as depicted in Figure 6. Figure 7 illustrates contour plots of the out-of-plane displacements in these three moments.

**Figure 8:**
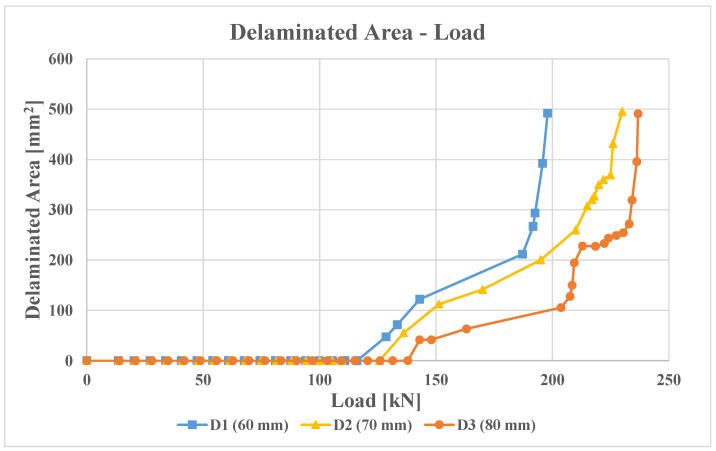
Comparison of delaminated area versus load for D1, D2, and D3 configurations under non-linear static analyses.

Furthermore, non-linear static analyses were conducted with the virtual crack closure technique to determine the delamination growth. Table 2 presents the onset delamination loads. It is evident that as the diameter of damage increases, the delamination initiation load is delayed. This phenomenon can be explained by evaluating the energy accumulated near the damage during the compression analysis. If the damage is minor, the possibility of deformation is reduced, and this energy must be released by detaching nodes between the laminate nodes. If the damage is extensive, the deformation possibility increases, as does the energy released during both deformation and detachment. Consequently, the onset delamination load is delayed. The damage growth versus load for D1, D2, and D3 configurations is reported in Figure 8. It can be observed that, as the damage propagates, the reaction loads increase, and the damage becomes unstable. Furthermore, for a given reaction load, the corresponding delaminated area is greater in the configuration with a smaller damage diameter.

### 4.2. Effects of Delamination Depth (Non-Linear Static Analyses)

The D4, D5, and D6 configurations have identical geometries with the sole distinction being the initial damage depth, which is 0.33 mm, 0.495 mm, and 0.66 mm, respectively. Table 3 presents the stiffness values for the three panels, which are found to be equal, thereby confirming that the depth of the damage does not influence global stiffness. Subsequently, these configurations were subjected to non-linear static analyses, with out-of-plane displacements calculated at the centre of the circular delamination. The instability loads, created by bending, are presented in Table 3. The formula for computing the critical load of a panel under compression posits that the buckling load is directly proportional to the minimum moment of inertia of the section. Consequently, the critical load of deflection increases in proportion to the minimum moment of inertia of the section. The results obtained from the conducted analyses corroborate this trend, with the D4 configuration exhibiting a buckling damage load of 37,900 N, the D5 configuration demonstrating a load of 47,351 N, and the D6 configuration, with the largest diameter, exhibiting the highest load of 60,773 N.

**Figure 9:**
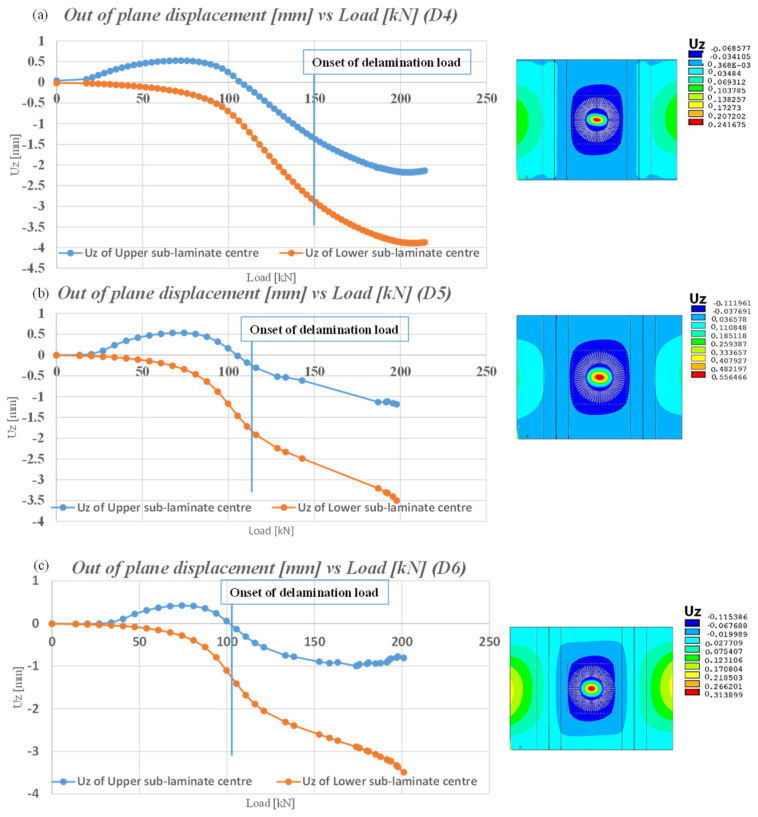
Out of plane displacement versus load for D4, D5, and D6 configurations.

Figure 9 illustrates the qualitative performance of the out-of-plane displacements in the centre of the circular delamination. The calculations are presented for the centre of the first layer of the upper laminate and on the last layer of the bottom laminate. The different stiffness of the sublaminates generates the deformations visible in Table 2. In particular, the D6 panel has a stiffer upper sublaminate, which leads to a more pronounced opening and greater divergence between the sublaminates.

**Figure 10:**
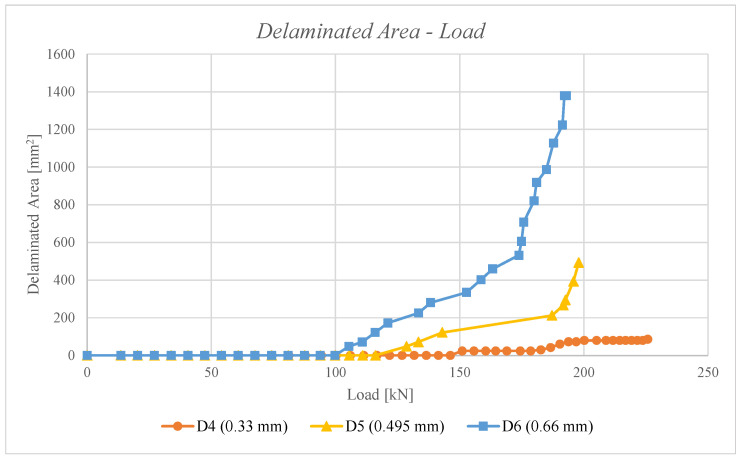
Comparison of delaminated area versus load for D4, D5, and D6 configurations under non-linear static analyses.

Furthermore, non-linear static analyses were conducted using the virtual crack closure technique to determine the delamination growth. As can be seen in in Table 3, the onset delamination loads are reported. It is evident that as the depth of damage increases, the delamination initiation load is brought forward. This phenomenon can be explained by evaluating the energy accumulation near the damage during the compression analysis. If the damage is deep, the possibility of deformation is lower, and this energy must be released by detaching nodes between the laminate nodes. In the event of superficial damage, the deformation possibility boosts, and the energy is released both in deformation and in detachment; hence, the onset delamination load is delayed. Figure 10 illustrates the damage growth versus load for the D4, D5, and D6 configurations. It can be observed that as the reaction loads increase, the damage propagation becomes unstable. Moreover, for a given reaction load, the corresponding delaminated area is greater for a configuration with deeper damage.

Table 4 provides a summary of the principal characteristics of all the configurations. It is noteworthy that, by fixing the delamination depth, an increase in the delamination diameter results in a reduction in the critical buckling load and an increase in the delamination initiation load (configurations D1, D2, and D3). Then, starting from the D1 configuration, which has been found to be the most critical in terms of delamination onset, a fixed diameter has been considered, while moving the delamination along the specimen thickness. These new configurations, namely D4, D5 (which correspond to D1 configuration), and D6 have been numerically analysed, and their mechanical behaviour has been compared. In particular, the relocation of the delamination near the surface has been observed to result in a reduction in the critical buckling load and a delay of delamination onset load. Conversely, a deeper delamination results in an increase in the critical buckling load and a decrease in the delamination initiation load.

### 4.3. Effects of Delamination Diameter (Fatigue Analyses)

**Figure 11:**
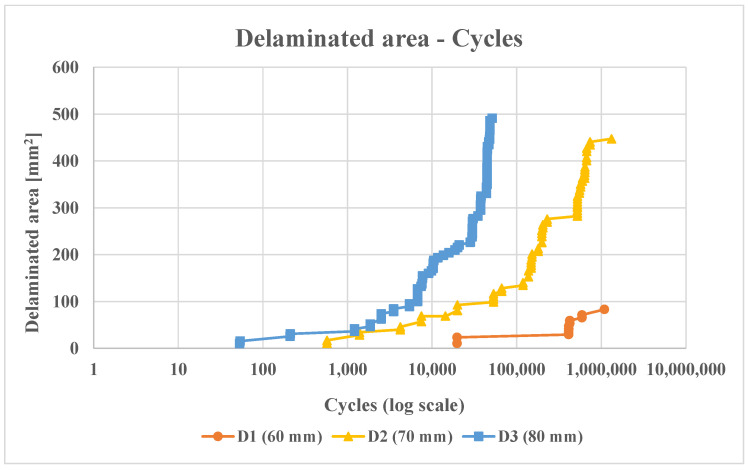
Comparison of delaminated area versus cycles for D1, D2, and D3 configurations under fatigue analyses.

In order to explore the influence of the delamination diameter on the propagation under fatigue loading conditions, fatigue analyses have been performed by applying a cyclic load to the structure ranging from 0 to 90% of the delamination onset load obtained from the non-linear static analyses. Figure 11 shows the evolution of the delaminated area as a function of the number of cycles during the fatigue analysis.

In the context of fatigue loading conditions, an examination of the delamination propagation as depicted in Figure 10 reveals a clear relationship between the delamination radius and the extent of damage. In particular, the analysis demonstrates that as the delamination radius increases, there is a corresponding increase in the propagated area of delaminations during a given fatigue cycle. This indicates a direct correlation between larger delamination radii and more pronounced damage propagation.

Moreover, it is apparent that configurations with larger delamination radii demonstrate increasingly unstable damage propagation with the progression of cycles. This is particularly evident in the comparative analysis of three configurations (D1, D2, and D3) during fatigue testing. Configurations D1 and D2 predominantly exemplify damage progression in accordance with the Paris law, which underscores that damage evolves in a predictable manner without immediately leading to static failure. It is noteworthy that the D1 configuration is the most damage-tolerant under fatigue conditions, exhibiting a stable propagation pattern with minimal deviation over increasing cycles. The symmetry in delamination growth is a defining feature of configuration D2. However, as the number of cycles increases, the system becomes unstable, indicating the presence of a critical threshold beyond which the damage propagation rate accelerates.

**Figure 12:**
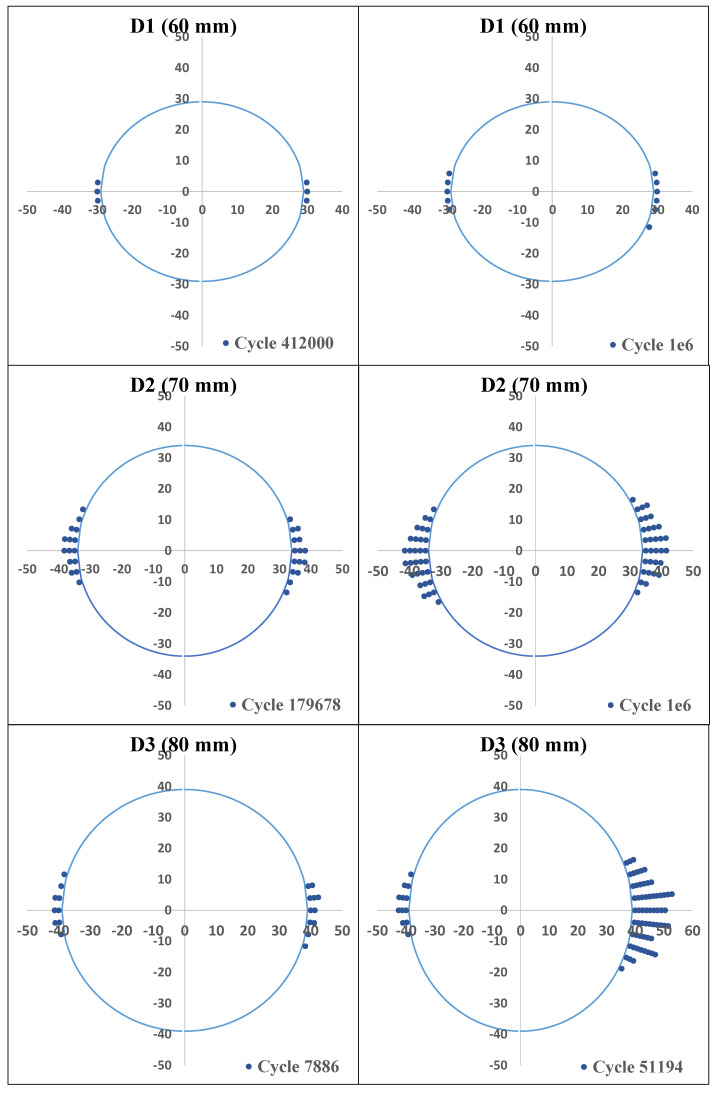
Delamination growth of configurations D1, D2, and D3, respectively.

In contrast, the D3 configuration, which is characterised by the greatest delamination radius, not only exhibits substantial damage growth at lower cycle numbers but also encounters static failures during the fatigue analysis. This dual occurrence of fatigue and static failures in D3 serves to illustrate the heightened instability in damage propagation when compared to the other configurations. Figure 12 provides further insight into the asymmetrical nature of damage propagation in D3 by illustrating the damage evolution across cycle numbers for each configuration. The occurrence of static fractures in D3 serves to exacerbate the aforementioned asymmetric propagation pattern, in stark contrast to the comparatively stable and symmetrical growth observed in D1 and D2.

The role of the Paris law in these observations is of great significance, as it provides a framework for understanding fatigue-driven delamination growth. The Paris law provides a means of describing the rate of crack growth per cycle as a function of the stress intensity factor range, which is instrumental in predicting the fatigue life of materials subjected to cyclic loading. In this study, the application of the Paris law enables a quantifiable assessment of delamination progression in configurations D1 and D2, where static failures are absent. However, the limitations of this approach are evident in D3’s unstable propagation, which serves to highlight the complexities introduced by larger delamination radii and static failures.

In conclusion, the results demonstrate the pivotal role of the delamination radius in determining the stability of damage propagation under fatigue conditions. The analysis of configurations D1, D2, and D3 offers valuable insights into the intricate relationship between fatigue cycles, delamination sizes, and damage growth dynamics, with significant implications for the design and evaluation of materials subjected to cyclic loading.

## 5. Conclusions

The objective of this study was to perform a sensitivity analysis of two geometrical parameters of stiffened composite panel delamination, in order to gain insight into delamination growth under static loading conditions. In this analysis, the delamination depth and its radius have been identified as relevant geometrical parameters.

A non-linear static analysis was initially conducted, and the onset load for damage propagation was calculated using the virtual crack closure technique (VCCT). The configuration exhibited a smaller delamination to propagate damage at an earlier stage than the others. This phenomenon is attributed to the accumulation of energy near the damage site; when the damage is smaller, the deformation capacity is reduced, leading to energy dissipation through the separation of nodes between the delaminated laminates. It was also observed that when the delamination is deeper, a similar effect occurs: the reduced deformation capacity of the panel results in energy dissipation through the separation of artificial delamination nodes.

Subsequently, an analysis of fatigue was conducted, focusing on the radius variation, with the delaminated area expressed as the number of cycles and the shape of delamination growth illustrated. The results demonstrate that the numerical methodology is capable of replicating the erratic propagation of delamination under cyclic loading conditions in composite material structures through a series of non-linear static analyses employing the VCCT method. Furthermore, this method is able to account for the rapid variation in delamination size associated with the reduction in bearing capacity of the structure during the unstable growth phenomenon.

## Figures and Tables

**Table 1:** Carbon fibre/epoxy resin material properties.

Property	Value	Description
$E_{11}$	122,000 MPa	Young’s modulus in the fibres’ direction.
$E_{22}=E_{33}$	6265 MPa	Young’s modulus in the transverse directions.
$G_{12}=G_{13}$	4649 MPa	Shear modulus in the 1–2 and 1–3 planes.
$G_{23}$	4649 MPa	Shear modulus in the 2–3 plane.
$\nu_{12}=\nu_{13}$	0.3008	Poisson’s ratio in the 1–2 and 1–3 planes.
$\nu_{23}$	0.02	Poisson’s ratio in the 2–3 plane.
$G_{Ic}$	$180\;J/m^{2}$	Mode I critical energy release rate.
$G_{IIc}=G_{IIIc}$	$1900\;J/m^{2}$	Mode II and Mode III critical strain energy release rate.
$t_{h}$	0.165 mm	Ply thickness.

**Table 2:** D1-D2-D3 stiffnesses, buckling local loads, and onset delamination loads.

ID Configuration	Stiffness	Local Buckling Load	Onset of Delamination Load
D1 (60 mm)	281,400 N/mm^2^	47,351 N	128,540 N
D2 (70 mm)	281,410 N/mm^2^	34,129 N	136,105 N
D3 (80 mm)	281,411 N/mm^2^	28,000 N	142,990 N

**Table 3:** D4-D5-D6 stiffnesses, buckling local loads, and onset delamination loads.

ID Configuration	Stiffness	Local Buckling Load	Onset of Delamination Load
D4 (0.33 mm)	281,419 N/mm^2^	37,900 N	150,964 N
D5 (0.495 mm)	281,400 N/mm^2^	47,351 N	128,540 N
D6 (0.66 mm)	281,411 N/mm^2^	60,773 N	105,370 N

**Table 4:** Comparison of stiffnesses, buckling local loads, and onset delamination loads of all configurations.

ID Configuration (Variable Diameter)	Stiffness	Local Buckling Load	Onset of Delamination Load
D1 (60 mm)	281,400 N/mm^2^	47,351 N	128,540 N
D2 (70 mm)	281,410 N/mm^2^	34,129 N	136,105 N
D3 (80 mm)	281,411 N/mm^2^	28,000 N	142,990 N
**ID Configuration** **(Variable Depth)**	**Stiffness**	**Local Buckling Load**	**Onset of Delamination Load**
D4 (0.33 mm)	281,419 N/mm^2^	37,900 N	150,964 N
D5 = D1(0.495 mm)	281,400 N/mm^2^	47,351 N	128,540 N
D6 (0.66 mm)	281,411 N/mm^2^	60,773 N	105,370 N

## Data Availability

The original contributions presented in the study are included in the article, further inquiries can be directed to the corresponding author.

## References

[1] Peng A., Deng J., Ren T., Wu D., Zhou G., Wang X. (2023). On damage behavior and stability of composite T-shaped stiffened panels under compression after impact considering impact locations. Thin Walled Struct..

[2] Zou J., Lei Z., Bai R., Liu D., Liu H., Huang X., Yan C. (2023). Damage evolution and failure mechanism of asymmetric composite laminates under low-velocity impact and compression after impact. Thin Walled Struct..

[3] Hu C., Xu Z., Chen D., Huang M., Cai C., Qiu J., He X. (2024). A novel integrated modeling strategy for predicting damage mechanisms and energy dissipation of composite stiffened structures under low-velocity impact and compression. Aerosp. Sci. Technol..

[4] Bai Y., Xu Z., Song J., Miao L., Cai C., Yang F., Wang R., He X., Hong Y., Dong X. (2020). Experimental and numerical analyses of stiffened composite panels with delamination under a compressive load. J. Compos. Mater..

[5] Raimondo A., Doesburg S.A., Bisagni C. (2020). Numerical study of quasi-static and fatigue delamination growth in a post-buckled composite stiffened panel. Compos. Part B Eng..

[6] Hiremath P., Viswamurthy S.R., Shettar M., Naik N., Kowshik S. (2022). Damage Tolerance of a Stiffened Composite Panel with an Access Cutout under Fatigue Loading and Validation Using FEM Analysis and Digital Image Correlation. Fibers.

[7] Diao X., Lessard L.B., Shokrieh M.M. (1999). Statistical model for multiaxial fatigue behavior of unidirectional plies. Compos. Sci. Technol..

[8] Whitworth H.A. (2000). Evaluation of the residual strength degradation in composite laminates under fatigue loading. Compos. Struct..

[9] Pantelakos S.G., Kyriakakis E.C., Papanikos P. (2001). Non-destructive fatigue damage characterization of laminated thermosetting fibrous composites. Fatigue Fract. Eng. Mater. Struct..

[10] Pantelakis S.G., Kyriakakis E.C. (1999). Fatigue damage of APC-2 composite assessed from material degradation and non-destructive evaluation data. Theor. Appl. Fract. Mech..

[11] Tserpes K.I., Papanikos P., Labeas G., Pantelakis S.G. (2004). Fatigue damage accumulation and residual strength assessment of CFRP laminates. Compos. Struct..

[12] Wimmer G., Pettermann H.E. (2009). Prediction of delamination growth in laminated structures loaded by quasi-static and cyclic loads. J. Compos. Mater..

[13] Krueger R. (2015). The virtual crack closure technique for modeling interlaminar failure and delamination in advanced composite materials. Numerical Modelling of Failure in Advanced Composite Materials.

[14] Russo A., Sellitto A., Palumbo C., Castaldo R., Riccio A. (2024). Parametric Investigation of Stiffened Panel Subjected to Compressive Loads: Influence of Initial Delamination Length on Damage Behaviour. Procedia Struct. Integr..

[15] Riccio A., Raimondo A., Scaramuzzino F. (2015). A robust numerical approach for the simulation of skin–stringer debonding growth in stiffened composite panels under compression. Compos. Part B Eng..

[16] Pietropaoli E., Riccio A. (2010). On the robustness of finite element procedures based on Virtual Crack Closure Technique and fail release approach for delamination growth phenomena. Definition and assessment of a novel methodology. Compos. Sci. Technol..

[17] Russo A., Riccio A., Sellitto A. (2022). A robust cumulative damage approach for the simulation of delamination under cyclic loading conditions. Compos. Struct..

[18] Russo A., Riccio A., Palumbo C., Sellitto A. (2023). Fatigue driven delamination in composite structures: Definition and assessment of a novel fracture mechanics based computational tool. Int. J. Fatigue.

[19] Aris P.C., Gomez M.P., Anderson W.E. (1961). A rational analytic theory of fatigue. Trend. Eng..

[20] Paris P.C., Erdogan F.A. (1963). Critical analysis of crack propagation laws. J. Basic Eng..

